# Performance Analysis of Reinforced Epoxy Functionalized Carbon Nanotubes Composites for Vertical Axis Wind Turbine Blade

**DOI:** 10.3390/polym13030422

**Published:** 2021-01-28

**Authors:** Yasser Elhenawy, Yasser Fouad, Haykel Marouani, Mohamed Bassyouni

**Affiliations:** 1 Mechanical Power Engineering Department, Port-Said University, Port-Said 42526, Egypt; dr_yasser@eng.psu.edu.eg; 2 Faculty of Applied Engineering, Muzahimiyah Branch, King Saud University, P.O. Box 2454, Riyadh 11451, Saudi Arabia; hmarouani@ksu.edu.sa; 3 Department of Chemical Engineering, Faculty of Engineering, Port Said University, Port-Said 42526, Egypt; m.bassyouni@eng.psu.edu.eg; 4 Materials Science Program, University of Science and Technology, Zewail City of Science and Technology, October Gardens, 6th of October, Giza 12578, Egypt

**Keywords:** polymer nanocomposite, vertical axis wind turbine, finite element analysis, ANSYS.

## Abstract

Synthetic materials using epoxy resin and woven Kevlar fiber nanocomposites were fabricated in the presence of functionalized multiwalled carbon nanotubes (F-MWCNTs). Kevlar-reinforced epoxy nanocomposites were designed to manufacture a small blade of vertical axis wind turbines (VAWT). It is important to estimate the deflection of the versatile composite turbine blades to forestall the blades from breakage. This paper investigates the effect of F-MWCNTs on mechanics and deflection of reinforced epoxy composites. The outcomes show that the mixing of F-MWCNTs with epoxy resin using a sonication process has a significant influence on the mechanical properties. Substantial improvement on the deflections was determined based on finite element analysis (FEA). The vortices from the vertical axis wind turbines (VAWTs) blades have a negative impact on power efficiency, since small blades are shown to be effective in reducing tip vortexes within the aerospace field. To support the theoretical movement of the VAWT blade, modeling calculations and analyzes were performed with the ANSYS code package to achieve insight into the sustainability of epoxy nanocomposites for turbine blade applications below aerodynamic, gravitational, and centrifugal loads. The results showed that the addition of F-MWCNTs to epoxy and Kevlar has a significant effect on the bias estimated by finite element analysis. ANSYS analysis results showed lower deflection on the blade using epoxy with an additional of 0.50 wt.% of MWCNTs-COOH at tip speed ratios of 2.1, 2.6, and 3.1.

## 1. Introduction

Today, the extent of consumption of nonrenewable fuel-related energy sources is very high. Solar energy, wind energy, hydroenergy, tidal energy, geothermal energy, and biomass energy are the foremost necessary kinds of renewable energy obtainable [1]. Wind generation has many advantages. It is clean, copious in nature, needs much less space, and finally, most importantly its extraction does not hurt the atmosphere [2,3]. Because of low density, low cost, affordable distinctive strength properties, and better energy recovery, researchers are currently concentrating totally on synthetic-reinforced polymer composites for fabrication of wind turbine blades [4,5].

Several synthetic polymer composites are used for wind turbine blade fabrication. glass, Kevlar, and carbon-reinforced matrix are commonly used. Combining Kevlar with epoxy matrix creates a composite that is durable, light, and dimensionally stable. Dielectric strength of Kevlar improves epoxy lightning damage resistance. Composite materials can provide higher stiffness in many cases and reduce the weight of finished parts. These materials may require unique manufacturing techniques to meet design requirements. Thermosetting and thermoplastics polymers can be used for such techniques. Thermoplastics are more viscous when compared to thermosets so impregnation is difficult in thermoplastics. Increasing temperature lowers viscosity, but in some polymers decomposition can occur before very low viscosity is obtained. Epoxy and vinyl ester are the most common matrices that are used to fabricate wind turbine blades. Various functionalized carbon nanotubes (CNTs) such as amino and carboxylated functionalized multiwalled carbon nanotubes (F-MWCNTs) are used to improve the mechanical and physical properties of epoxy [6,7]. The vacuum infusion process (VIP) offers advantages over the conventional resin transfer molding (RTM) process [5]. Vertical axis wind turbines (VAWTs) have a promising market and have recently received a great research interest. Several studies have implemented the simulation technologies in order to analyze and develop new designs for VAWTs. This includes both the aerodynamic aspects [8] and the structure aspect [9,10,11] of the VAWTs’ design. The performance of a turbine could vary depending on the material designated. Composites are the most favored material for the production of blades because of their varied properties. Muhammed et al. [12] investigated the feasibility of a nanocomposite for use in wind turbine blades. It is produced by the manual laying method to add different percentages of montmorillonite nanoclay to the AW 106 Epoxy/E-Glass composite fiber. From the test results, the composite with 1% montmorillonite showed higher tensile stress and optimal hardness. This proves that the composite with 1% montmorillonite is appropriate for turbine blade applications. Stiffness and weight are major sources of design concern in the fabrication of wind turbine rotor blades, and an often neglected structural concern is blade wear properties. Under loading conditions, the interaction between the rotor blade and air current (consisting of air molecules and other small-sized airborne matter mixed in the flow) can be turbulent, and can generate a significant amount of frictional contact at the surfaces of the blades. This process is akin to sand blasting the surface of a work piece that leads to material loss, thus exposing underlying layers with processing defects hidden within the volume of the work piece.

Muhammed et al. [13] examines the feasibility of nanocomposites-supported SiO_2_ and Al_2_O_3_, especially for the utilization of wind turbine blades. The composites were made by a hand-applied procedure that changes the share of nanomaterials. Results showed that the nanocomposite with 1% Al_2_O_3_ confirmed extraordinarily good tensile strength and fatigue strength with most excellent hardness. The results of the ANSYS evaluation showed that the nanocomposite with 1% Al_2_O_3_ worked properly under edge and flap loads, which is very acceptable for a long lifetime of wind turbine blades.

The assessment of the structural integrity and harm of the lengthy wind blade under excessive load conditions was studied [14]. The blade was first analyzed for excessive loading conditions, wherein aerodynamic buoyancy forces precipitated a large deflection of the flap, while gravitational loading caused bending from the edge toward the trailing edge. Shear bands additionally revel in better stresses, which may be decreased through increasing the number of biaxial layers within them. In addition, the nearby buckling in the pores and skin was examined by a linear buckling evaluation, which showed the nearby buckles of the pores and skin on the equivalent area, which were discovered by the nonlinear evaluation of the large deflection. The results showed that the tip deflection was 17.9% of the blade length, akin to different comparable systems.

A horizontal axis five Megawatts (MW) wind turbine blade was designed and a calculation analysis was carried out. Ansys Fluent was used for the analysis and finishing. It was observed that the utmost momentum was formed at three different entry velocities: 3.5, 12, and 24 m/s at 80° of blade attack angle (Amjith and Bavanish [15]).

As wind turbines expand, suitable regulations are necessary to assure reliable operation and maintenance [16]. To make certain the blade conforms to the planning, it is not permitted for the blade tip deflection and technical stress to exceed the permitted limits of 5.56 m and 0.562%. A computational aerodynamic analysis was performed with a computational fluid dynamic (CFD) model, depending on the wing design and running conditions; both fibers met the structural requirements below regular running conditions. A 45% reduction in wing tension was found when basalt fibers were used rather than glass fibers, and a 68% decrease in overall deformation was achieved for an essential load case of 39 m/s when combined basalt fibers were used. Structural and most energy generation requirements for this wind turbine design were met [17].

The aerostructure multidisciplinary optimization system was performed for the SERI-8 wing using the Q blade for 2D aerodynamic evaluation. Therefore, the ANSYS bench for 3D aerodynamic and structural evaluation was carried out [18]. A new methodology for reducing the weight of blades for compound wind turbines was applied by a combination of evolutionary and topological optimization schemes in an alternating mode. First, the most appropriate distribution of the laminate within the outer shell of the blade was explored by the use of genetic algorithms and assuming that the shear bands are completely dense. In each case, the blade was exposed to an intense load situation with constraints of peak displacement, stress, normal vibration frequencies, and buckling phenomena. As a further feature, the method integrates the inverse finite element technique to restore the aerodynamically efficient shape of the blade while operating in an ordinary load situation, and to increase the protection margin of the tower in an intense load situation. Results display a large financial savings of as much as 23% and a large increase within the tower clearance margin of safety. Condruz et al. [19] studied the experimental model of wind turbine blades with a counter-rotating vertical axis. The material was selected on the basis of the requirements placed on turbine blades with a vertical axis in terms of mechanical properties, high rigidity, low density, and ecological compatibility. The difference between the blades is due to the length of the chord.

Song et al. studied aerodynamic contour layout and overall performance evaluation of wind turbine blades as a critical part of wind turbine layout and application [20]. According to the theory of transformation of cosmic coordinates, the spatial coordinates of the blade elements were calculated. Based on a dynamic analysis of the performance of a compound blade, a method for modeling the combination of Solid Works with ANSYS was planned, referring to the actual layer structure. This study was successfully applied to the production of blades of composite wind turbines with an output of 20 kW. Results show significant differences between loading approximation methods for both deflection and strain results. The scope of tip rotation of 4.88° for the CFD approach is somewhat underestimated by the uniform pressure distribution and much lower for the tip load approaches [21]. The deflection was significantly overestimated by the point load approach. The first point load approach had an extreme tip deflection and maximum compressive stress results. With delamination being the principal reason for malfunctioning turbine blades, fatigue loads were recognized as one of the most important reasons for damage. The article provides a review of fatigue damage experienced in wind turbine blades and discussed the factors affected by fatigue loads [22]. The fluid flow of the composite material wind turbine blade was analyzed by Sathish [23]. Materials together with polyesters and epoxy were used to make the composite, which are used within the turbine blade. The overall performance of the proposed blade was analyzed using CFDs within the Ansys platform. Performance under different conditions such as temperature, pressure, and speed variation were investigated. Simulation evaluation gives higher overall performance and the proposed composite material can offer higher efficiency in power generation. Thus, the proposed composite material becomes suitable for the manufacture of wind turbine blades. The current trend aims to synthesize functionalized polymer nanocomposites to manufacture wind turbine rotor blades for additional structure reinforcement with strong, lightweight mechanical properties and cost-effective materials. Wind turbine blades are affected by a variety of loading conditions, which under certain circumstances can rapidly destroy the blade and prevent power generation from the unit. Most of these severe loading conditions are unavoidable, but the effects which they have on the turbine blade can be controlled and mitigated by tactful use of engineering. This study aims to mitigate blade deflection due to loading on the blade structure. These events result from the interaction between, on the one hand, particulate-laden gust winds and rain, and on the other, the turbine blade material surfaces. It is a progressive process that can be significantly slowed by selecting good mechanically resistant materials for turbine blade fabrication. Nevertheless, a large challenge is that all of these modes of damage are not readily detectable, since the damage may not occur on external surfaces and therefore not visible. For example, wrinkles may lead to thick composite pieces that lead to the formation of compression loss and delamination. Cracks and delamination will also begin to process information such as ply-drops that create a concentration of tension locally. The objectives of this study are to (i) prepare a well-dispersed F-MWCNTs-epoxy mixture using a sonication process to overcome the agglomeration of MWCNTs; (ii) identify, develop, and test nanocomposite materials that can withstand a combination of environmental conditions; (iii) verify the proper mechanical and physical testing for detecting flaws, cracks, and voids of wind turbine blades exposed to dynamic stresses; and (iv) carry out a simulation using Ansys to study the deflection of wind blades at different wind speeds. In particular, this research aims to evaluate the Kevlar/epoxy functionalized CNTs composites for high performance wind turbines, such as those used in onshore and offshore wind farms.

## 2. Materials and Methods

Mixing MWCNTs with epoxy resin using sonication technique was applied to improve the dispersibility of CNTs. Synthetic fiber-epoxy composites were manufactured using vacuum infusion process. Surface morphology of fracture surface was investigated using scanning electron microscopy (SEM) to study the matrix-fiber interfacial strength. Tensile properties were measured to determine the ultimate tensile strength. Computational fluid dynamics simulation was carried out based on mechanical testing results.

### 2.1. Materials Characterization

Five samples of unmodified epoxy and F-MWCNTs mixtures were synthesized for nanocomposite fabrication. Nanocomposite materials behave differently depending on the work plane, and in order to get valid simulation results, the properties of the materials must be known. Samples comprising F-MWCNT/epoxy/Kevlar laminates were prepared. Unmodified and functionalized (—COOH) grade multiwalled carbon nanotubes were used. Carbon nanotubes were received from Grafen Co., Ankara, Turkey. The specifications of the MWCNTs are given in Table 1. AMPRO™ Slow Hardener resin, purchased from Gurit, Isle of Wight, UK, is a slow hardener-epoxy resin. Physical and mechanical properties of epoxy are given in Table 2.

Bisphenol-A diglycidyl ether-based epoxy is mixed with a hardener in a 100:29 ratio based on the supplier data sheet. Neat epoxy has a tensile strength of 48.1 MPa and modulus of elasticity 2.7 GPa if cured at room temperature for 24 h. Glass transition temperature after complete curing is 45 °C. Specifications of matrix and synthetic fiber are listed in Table 3.

**Figure 1:**
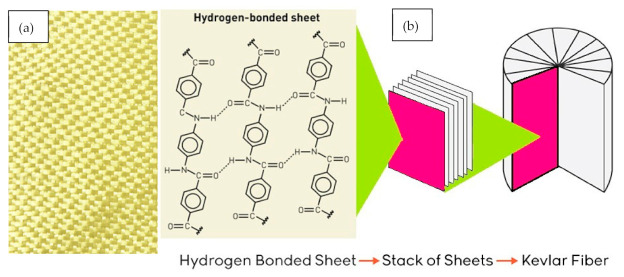
(**a**) Kevlar cloth fabric plain 175 g and (**b**) structure of Kevlar fiber.

Kevlar fibers are high modulus fibers with applications in polymers reinforcements, as shown in Figure 1a, and based on poly (*p*-phenylene terephtalamide). Fibers are produced in a solution extruder where fiber strength can be controlled. The phenyl groups of the terephthalic segment and *p*-phenylene segment make angles of −30° and 38° with respect to the chain backbone. Two different models have been proposed for the supermolecular structure of Kevlar fibers. The first model proposes that the molecule’s hydrogen bond together to form sheet along the (100) crystal plane, as shown in Figure 1b. These sheets then stack around the fiber axis with (010) crystal direction pointing radially outward. The second model proposes that the fiber structure is composed of cylindrical surfaces sequentially arranged coaxially [24].

### 2.2. Fabrication of Kevlar-Reinforced Epoxy F-MWCNTs Nanocomposites 

Carbon nanotubes were pretreated with acetone in an ultrasonic bath at room temperature for 30 min to separate the nanotube bundles to ensure greater dispersion. Acetone was then evaporated from the blends by holding the samples for 1 h in a vacuum oven at 70 °C. To improve the dispersibility of MWCNTs in epoxy resin, the temperature of the resin was raised to 50 °C for 2 h to reduce the resin viscosity. The unmodified and F-MWCNTs were added at different percentages (0.1 and 0.5 wt.%) and mixed using ultrasonic technique. A titanium alloy probe was immersed in the epoxy/MWCNTs mixture.

**Figure 2:**
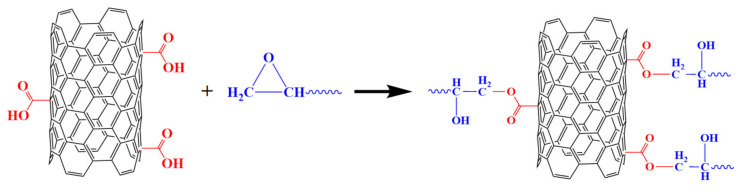
Schematic illustration of the nanocomposite structures of functionalization MWCNTs and epoxy.

In functionalized MWCNTs, carboxyl groups serve as proton donors in the epoxide ring opening reaction, leading to enhanced polymerization near the surface of nanotubes and increased interfacial bonding, as shown in Figure 2.

**Figure 3:**
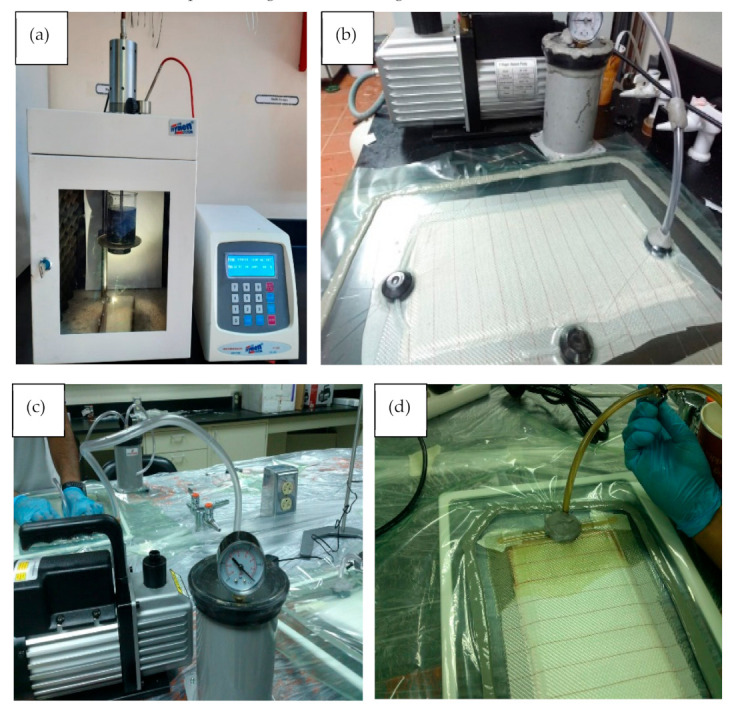
Synthesis and fabrication of Kevlar/epoxy/MWCNTs nanocomposite: (**a**) mixing epoxy/MWCNTs using sonication technique, (**b**) VIP tools, (**c**) evacuation process, and (**d**) fiber impregnation by the epoxy/MWCNTs mixture.

The ultrasonic generator transforms the electric current into ultrasonic energy of 20 Hz. Elastic distortion was induced by transducer as alternate voltage transduces mechanical vibration where cavitation and a multitude of microbubbles are formed. This technique allows the epoxy/MWCNTs mixture to undergo intense shaking and vibration, as shown in Figure 3a. The temperature of the MWCNTs/resin was maintained at 75 °C during the sonication process, in order to decrease resin viscosity and allow for better dispersion of the MCNTs into the epoxy resin.

Kevlar-reinforced epoxy CNTs were prepared using VIP technique. Figure 3b,c shows the tools and materials used for VIP technique. To prepare the fiber layers, the fiber was cut into a 25 × 25 cm^2^ sheet. Seven layers of Kevlar fiber were placed on a clean glass plate. Care was taken during fiber cutting due to the sensitivity of the material. Fibers could be very easily detached or disoriented from their original places, which would affect the flow of the resin during infusion and might undermine the mechanical properties of the final laminate. All holes were sealed using tacky tape. The vacuum pump was then switched on to start the evacuation process. It was allowed to evacuate for a while before resin infusion to ensure complete evacuation of the cavity and to increase the compaction of the fiber sheets, as shown in Figure 3d. The vacuum pump used in this study was an Easycomposite, Stoke-on-Trent, UK, 14 mbar. Time of evacuation ranged from 30 to 90 min. Resin and hardener were mixed thoroughly. Mixing lasted until no clouding was visible in the mixing container. Special attention was paid to the walls and the bottom of the mixing container to avoid residuals. A hole was made in the vacuum bag directly above the hole in the foam piece and the inlet pipe was inserted as quickly as possible to avoid air entry into the cavity after evacuation. A tacky tape was then placed over this hole to prevent any leakage. The mixed resin was placed in the resin reservoir and then the other end of the inlet pipe was inserted inside the resin reservoir. The fibers were impregnated by the resin/MWCNTs mixture at the flow front. The inlet pipe was clamped again when the flow front reached a length of 25 cm from the inlet. The vacuum pump was kept working until full curing was achieved.

The pressure from the vacuum pump provides both the driving force for the epoxy/MWCNTs mixture to flow inside the cavity and the compression force to compact the preform to the Kevlar volume fraction. The vacuum was left for 30 min until the resin had completely gelled. The Kevlar/epoxy/MWCNTs composites were cured at room temperature for one day. Five samples (KR0, KR1, KR2, KR3, and KR4) were prepared using a matrix made of neat epoxy, unmodified MWCNTs 0.1% and 0.5%, and COOH-functionalized MWCNTs 0.1% and 0.50%, as listed in Table 4.

### 2.3. Testing of Kevlar/Epoxy Functionalized MWCNTs Composites

Mechanical testing and surface morphology were carried out to study the effect of unmodified and F-MWCNTs on Kevlar-reinforced epoxy.

#### 2.3.1. Mechanical Testing

Mechanical tests were carried out on Kevlar/epoxy/F-MWCNTs nanocomposites to measure the static properties. Multidirectional Kevlar-reinforced epoxy specimens were fabricated according to ISO 527-4 and tested at a tensile speed of 2 mm/min. For each type of composite, the number of specimens used for tensile tests was five. Average values of tensile strength were reported and addressed for Kevlar epoxy composite materials.

#### 2.3.2. Surface Morphology

The morphology of the fracture surface of Kevlar-reinforced epoxy nanocomposites were investigated using a field emission scanning electron microscope (FESEM, FEIVerios 460L). Accelerating voltages of 5 and 20 kV were applied. A gold sputter coating of several nanometers was applied on tensile fracture surfaces.

### 2.4. Wind Turbine Model

**Figure 4:**
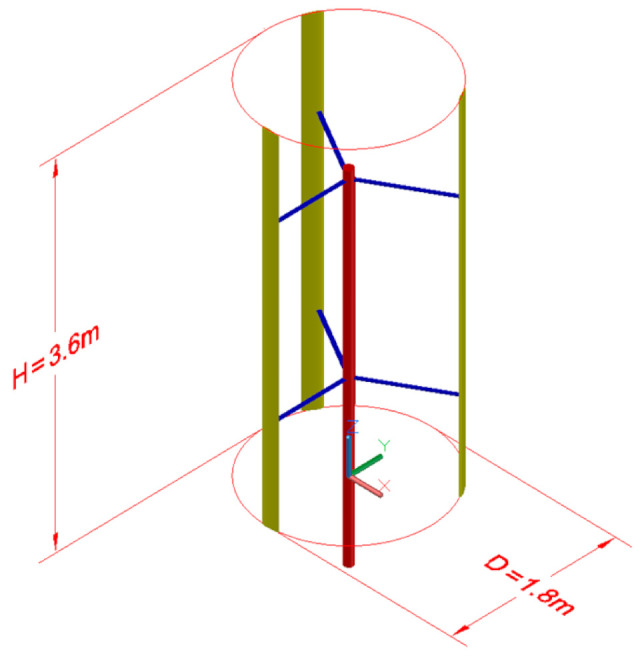
Three-dimensional geometry model of the optimized VAWT.

**Figure 5:**
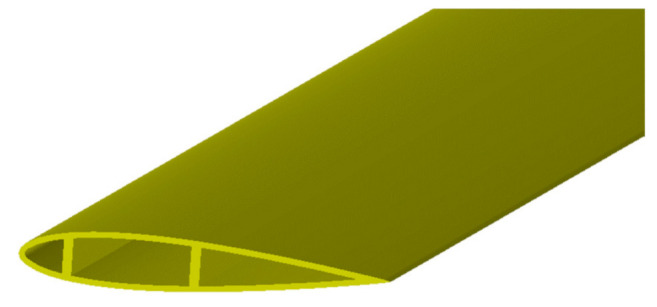
Three-dimensional geometry model of the optimized VAWT.

The VAWT geometry and its operating conditions were selected based on an optimization study. The optimized VAWT was a three-bladed VAWT with a 1.8 m diameter. Each blade followed the NACA20015 airfoil profile and had a span of 3.6 m. This gave an aspect ratio of two based on the ratio between the turbine height and diameter. At the optimal conditions, the turbine rotated at 20.222 rad/s when the wind speed was 7 m/s. The main parameters of the optimized turbine are presented in Table 5. Figure 4 illustrates the 3D geometry and the main dimensions of the optimized VAWT. Figure 5 shows the shape of the blade cross section, which includes two shear webs.

The tip speed ratio (TSR), which defines the relative velocity of the wind and the velocity of the blade, is presented in Equation (1):

(1) $$TSR=\frac{\Phi r}{U_{w}}$$

where Φ is rotor speed (rad/s), *r* is radius (m), and $U_{w}$ is wind speed (m/s).

### 2.5. Finite Element Analysis Modeling

The FEA modeling of the optimized VAWT with composite blades was carried out using the ANSYS Static Structural module [25], which is commonly used FEA modeling software. The FEA modeling procedure was applied to the optimized VAWT geometry. This section includes a detailed description of the geometry, meshing, materials, and boundary conditions that were implemented in the FEA model.

#### 2.5.1. Geometry

The geometry of the modeled VAWT includes the shaft, three blades, and six supporting struts. The geometry was created in Autodesk’s AutoCAD software (California United States) based on the information provided in Reference [24]. The shape of the VAWT geometry is illustrated in Figure 4.

#### 2.5.2. Material Properties

In the FEA model of the proposed VAWT, five different types of composite materials were considered. More details about the materials used and their properties are presented in Section 2.1.

#### 2.5.3. Finite Element Analysis Mesh

**Figure 6:**
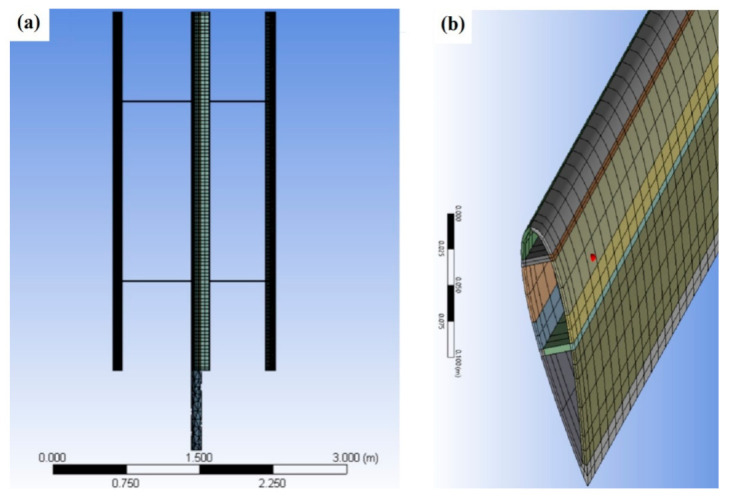
Finite element analysis (FEA) mesh: (**a**) blade and (**b**) close-up view of the blade tip.

**Figure 7:**
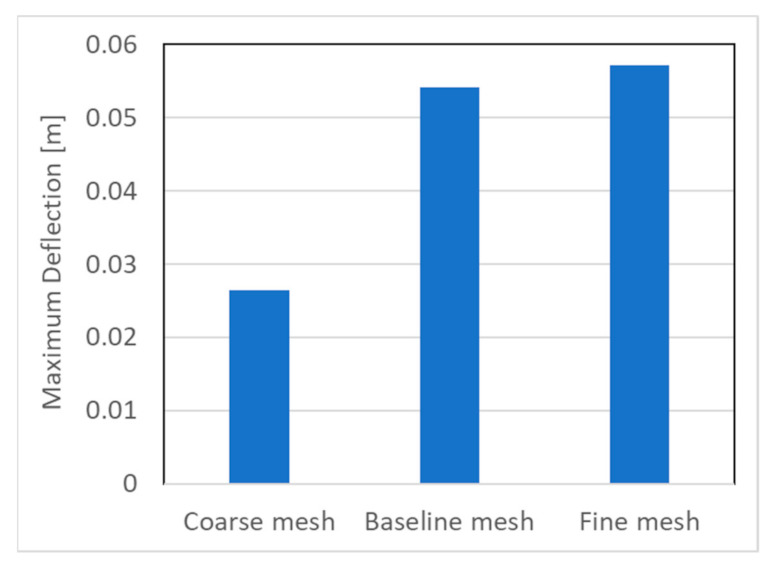
The effect of the mesh size on the predicted maximum deflections for the highest rotational velocity of 24.11 rad/s and using KR1 material.

A structured mesh topology was utilized for the meshing of the turbine blades. Figure 6a shows the baseline mesh for the whole turbine while Figure 6b illustrates the structured mesh for the turbine blade. A mesh sensitivity study was carried out in order to select the appropriate mesh that provides a good accuracy and a reasonable computational cost. Three meshes were constructed for the mesh sensitivity analysis; these include the coarse mesh with 32,198 nodes, baseline mesh with 94,809 nodes, and fine mesh with 135,317 nodes. More details about these meshes are available in Table 6. Figure 7 shows the effect of the mesh size on the predicted maximum deflections for the highest rotational velocity of 24.11 rad/s and using KR material. It is observed that the predictions of the baseline and the fine meshes show good agreement while the coarse mesh has a considerable underestimation. Therefore, the baseline mesh with 94,809 nodes and 19,935 elements was deemed as the appropriate mesh and used in the further tests.

#### 2.5.4. Boundary Conditions

The VAWTs were subjected to several loads and these included the aerodynamic, gravitational, and centrifugal loads. The current study focused mainly on the effect of the different structure materials and their properties on the maximum deflections of the blades at different centrifugal loads. These centrifugal loads corresponded to three different tip speed ratios (TSRs), including 2.1, 2.6 (optimal TSR), and 3.1, corresponding to 16.33, 20.22, 24.11 rad/s. These centrifugal loads were applied to the VAWT rotor in ANSYS Static Structure by imposing the relevant rotational velocity. In addition, a cylindrical support was applied to the turbine shaft.

#### 2.5.5. Solve and Postprocessing Results

ANSYS Static Structure software (Southpointe, 2600 Ansys Drive Canonsburg, Pennsylvania, USA) was utilized for the solution and postprocessing of the results. This software can perform different structural analyses, including static analysis and modal analysis. Several results can be extracted, such as the blade deformation and stress distribution. In the current study, the blade deformation was extracted from the static structure simulations.

## 3. Results and Discussion

### 3.1. Mechanical Testing

**Figure 8:**
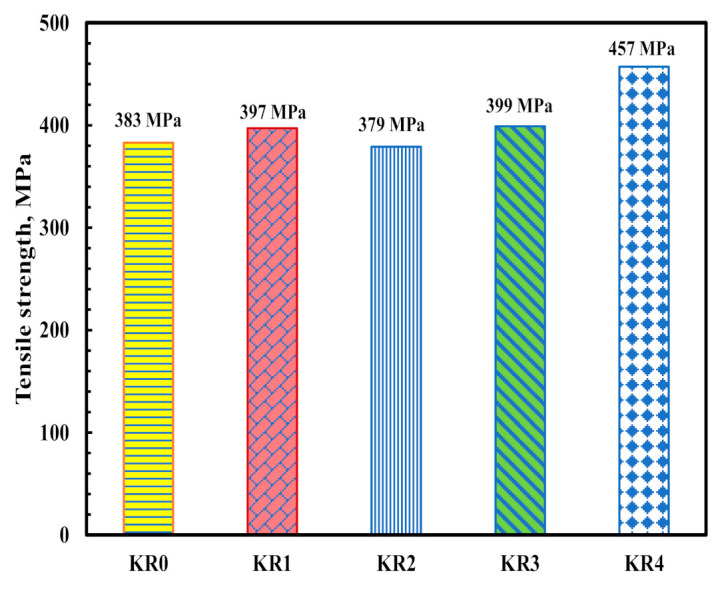
Effect of MWCNTs and F-MWCNTs on the mechanical properties of Kevlar-reinforced epoxy.

**Figure 9:**
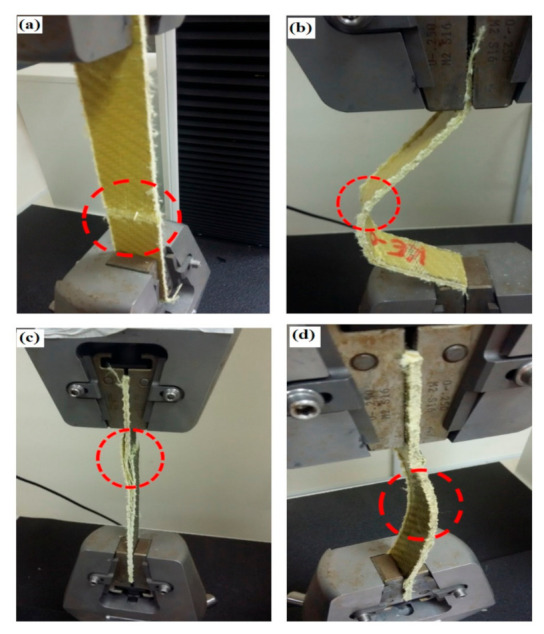
Mechanical testing: (**a**) Kevlar-reinforced epoxy under tension, (**b**) Kevlar-reinforced epoxy under compression, (**c**) Kevlar/epoxy MWCNTs (0.5 wt.%), and (**d**) Kevlar/epoxy F-MWCNTs.

Tensile strength of Kevlar-reinforced epoxy improved by 4% and 19% in the presence of F-MWCNTs (1%) and (5%), respectively. Figure 8 shows the tensile strength at different CNTs loadings. F-MWCNTs showed remarkable improvement in tensile strength of Kevlar-reinforced epoxy. Mechanical properties of neat epoxy were improved significantly by embedding synthetic Kevlar fiber. Nevertheless, the weak chemical interaction between the organic matrix and synthetic fiber can lead to composite delamination [26,27,28,29]. Delamination process is initiated at the boundary of structural elements. This interaction resulted in a chain of compression fiber failure throughout the structure. Figure 9a shows the fracture of Kevlar-reinforced epoxy under tensile stress. It was noticed that Kevlar-reinforced epoxy composite does not show delamination under compression stress, as presented in Figure 9b; however, the mechanical strength of KR0 is much less than Kevlar-reinforced epoxy-FMWCNTs. The number of delaminations that occurred in the specimen location on Kevlar-reinforced epoxy unmodified MWCNTs (0.5%) edges refers to low interfacial force between epoxy and MWCNTs. Delamination can be observed clearly under compression stress, as shown in Figure 9c. Because of the high affinity of CNTs, they create small clusters and influence the texture of polymer composites. Compared to those in a distributed state, tube-to-tube interactions have decreased the physical characteristics. This force entangles the CNTs triangular lattice structure and creates a bundling effect called agglomeration. CNTs agglomeration influences the aspect ratio and filler MWCNTs distribution as the aspect ratio is expected to decrease. Figure 9d shows a significant improvement in the interfacial force between matrix and fiber. This result with no delamination was obtained in the presence of functionalized MWCNTs. This can be a result from an even distribution of CNTs in the epoxy resin and a strong interface for epoxy-reinforcement. The surface of CNTs is extremely tortuous and greatly influenced by the chemical treatment. Potentially, carboxylic MWCNTs have reduced agglomeration. Fiber-reinforced polymers (FRP) are mostly used in wind turbine blades due to their high strength-to-weight and stiffness-to-weight ratios. One of the common failures in FPR laminates is delamination, which can result from manufacturing process defects or impacts from external forces. Delamination is an area of poor bonding between layers. Many factors can cause delamination, e.g., air traps, inadequate resin infusion, or production faults. Delamination lessens the compression strength of wind turbine blades and can induce local buckling in laminated structures [30,31,32]. Laminated composites containing delamination can buckle at low compression load levels depending upon the shape and size of delamination. Wind turbines fabricated using FRP and delamination can cause disastrous failure under different loading conditions [33].

### 3.2. Surface Morphology

**Figure 10:**
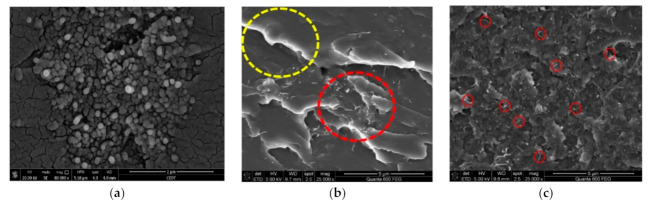
SEM image of (**a**) agglomoration of unmodified MWCNTs (0.5 wt/wt.%), (**b**) nonhomogenous dispersion of unmodified MWCNTs in epoxy resin, and (**c**) good dispersion of F-MWCNTs using ultrasonic mixing technique.

Figure 10 shows the SEM micrographs of woven Kevlar/epoxy composites in the presence of MWCNTs. It can be seen by close observation in Figure 10a,b that there is agglomeration of unmodified MWCNTs 0.5 wt.%, thus confirming the poor interfacial adhesion between Kevlar fibers/epoxy. The dispersibility of F-MWCNTs has been enhanced remarkably in epoxy, as shown in Figure 10c. The improvement in the dispersibility of carboxylated MWCNTs in epoxy can be attributed to several factors, including binding energy, packing density, and steric hindrance of F-MCNTs [34].

### 3.3. Finite Element Analysis

**Figure 11:**
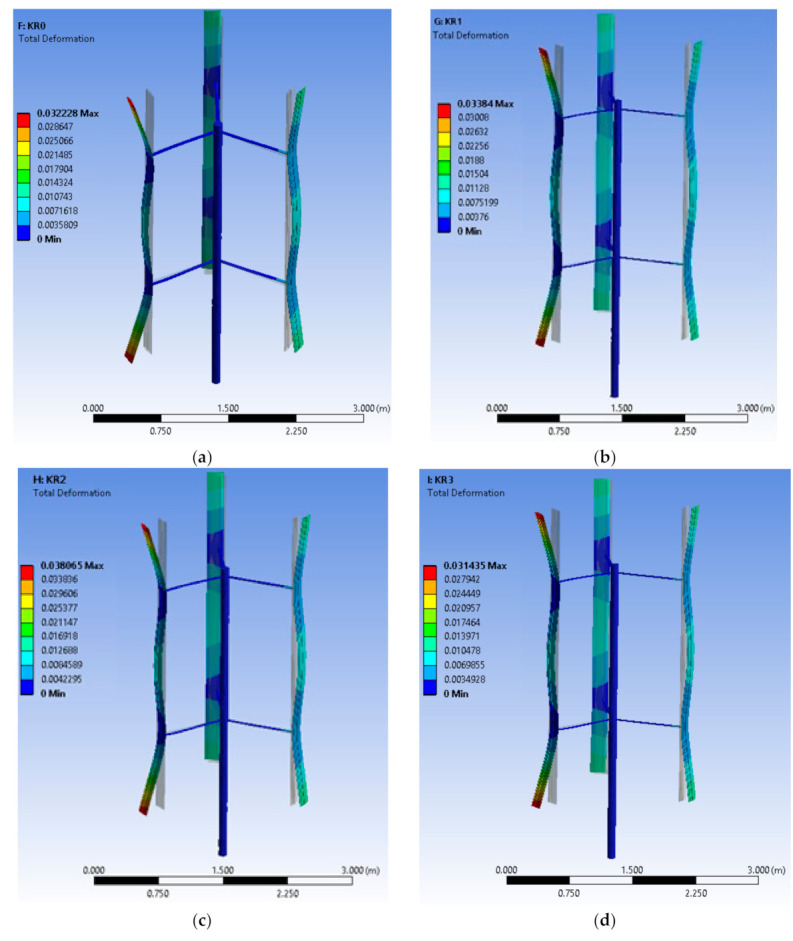

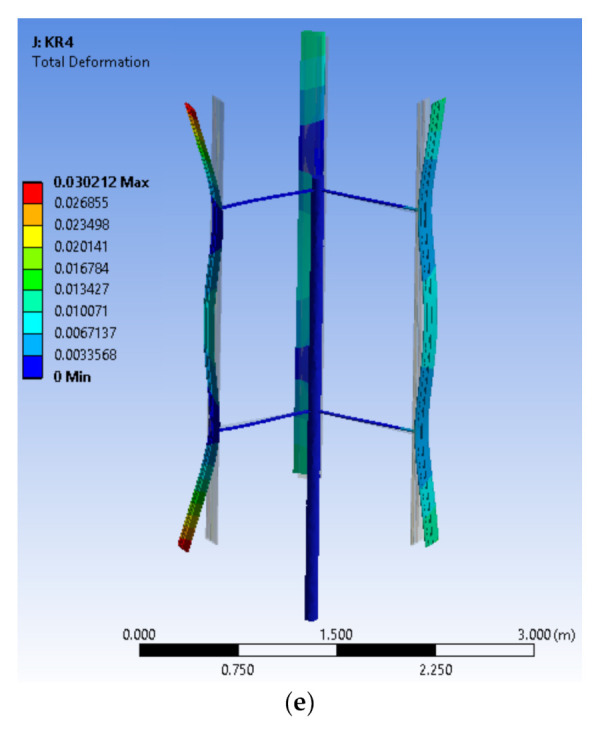
Graphical representation of the deformation of the turbine blades with (**a**) KR0, (**b**) KR1, (**c**) KR2, (**d**) KR 3, and (**e**) KR 4 material, at the optimal TSR of 2.6.

**Figure 12:**
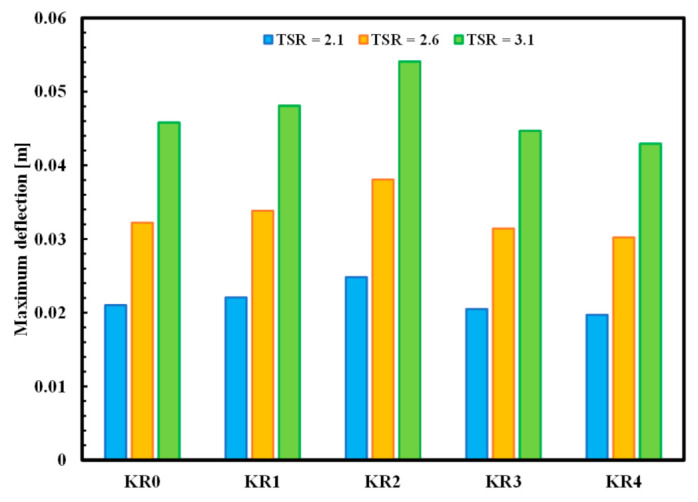
The maximum deflection of the blades at different TSRs for different KR-type materials.

Based on the method discussed in Section 2, the FEA structure analysis of an optimized VAWT was performed. Figure 11 shows a graphical representation of the deformation of the turbine blades with KR0, KR1, KR2, KR3, and KR4 material at the optimal TSR of 2.6. It is observed that the maximum deflections occur on the blade tips. In addition, high defections are observed at the blade midspan. Several tests were carried out in order to study the effects of implementing different materials on the maximum deflections at different centrifugal loads. Figure 12 shows the maximum deflection of the blades at different TSRs for different KR-type materials. KR2 showed the maximum deflection (0.054 m) at TSR 3.1. High deflection of KR2 can be attributed to the agglomeration of unmodified MWCNTs at high loading (0.5 wt.%). Deflection study using ANSYS is in good agreement with the surface morphology and mechanical testing results.

## 4. Conclusions

In this study, unmodified MWCNTs and functionalized MWCNTs-COOH were incorporated into epoxy in the presence of Kevlar fiber. The effect of F-MWCNTs and Kevlar fibers on the mechanical and physical properties of epoxy was studied in detail. Failure mechanisms of wind turbine blades depend upon materials or structural defects, and manufacturing process. Many factors like deflection of the wind turbine blades can cause delamination damage that can lead to blade failure. Tensile strength and surface morphology analyses showed that good dispersibility of F-MWCNTs (0.5 wt.%) was achieved using sonication process. Poor distribution and low mechanical strength of Kevlar/epoxy composites were observed in the presence of high CNTs load of unmodified MWCNTs (0.5 wt.%). The Ansys study showed that maximum deflection of Kevlar-reinforced epoxy was successfully decreased to 14% in the presence of F-MWCNTs (0.5 wt.%) at TSR 3.1.

## Figures and Tables

**Table 1:** Physical and chemical properties of multiwalled carbon nanotubes (MWCNTs).

Property	Value
Average diameter (nm)	9.5 nm
Length (µm)	0.5–1
Purity (wt.%)	90% MWCNTs 10% Metal oxides
Surface area (m^2^/g)	250–300
Thickness (nm)	~3
Bulk density (kg/m^3^)	215
Specific surface area (m^2^/g)	~25
Zeta potential (mv)	−30

**Table 2:** Physical and chemical properties of functionalized MWCNTs.

Property	Value
Outside diameter (nm)	10–30 n
Purity (wt.%)	>90
Length(um)	10–30
Surface area (m^2^/g)	250–300
Tapped density (kg/m^3^)	140
—COOH content (wt.%)	~1.5

**Table 3:** Physical and chemical properties of epoxy and Kevlar fiber.

Parameter	Epoxy (AMPRO™ Slow Hardener)	Kevlar 49
Tensile strength (MPa)	48.1	3600
Tg (°C)	45	455
Bulk density kg/m^3^	1120	1440
Hardness (HV)	15	27
Young’s modulus (GPa)	2.7	127
Viscosity (cP)	800	-

**Table 4:** Specification of Kevlar/epoxy MWCNTs nanocomposites samples.

Sample	Matrix	MWCNTs (wt.%)	MWCNTs	Reinforced	Kevlar Mass Fraction	Processing
KR0	Epoxy	0	0	Kevlar	0.63	VIP
KR1		0.1	Unmodified			
KR2		0.5	Unmodified			
KR3		0.1	–COOH			
KR4		0.5	–COOH			

**Table 5:** Main parameters of the optimized VAWT.

Parameters	Values	Units
Number of blades	3	-
Rotor radius, r	0.9	m
Blade length	3.6	m
Rated wind speed, $U_{w}$	7	m/s
Rated rotor speed,Φ	20.222	rad/s
Airfoil	NACA0015	

**Table 6:** Details of the different meshes used in the mesh sensitivity study.

	Coarse Mesh	Baseline Mesh	Fine Mesh
Nodes	32,198	94,809	13,5317
Elements	6692	19,935	26,672
Maximum Deflection [m]	0.0265	0.0541	0.0571

## Data Availability

The data presented in this study are available on request from the corresponding author.
